# Metapath2vec and attention-driven heterogeneous graph learning for prioritizing TCM-derived small molecules in gastric cancer

**DOI:** 10.1186/s13020-026-01431-y

**Published:** 2026-06-03

**Authors:** Nengquan Sheng, Jinran Liu, Wen Fang, Caijie Zheng, Yinuo Ma, Yang Sun, Hongqi Chen

**Affiliations:** 1 Department of General Surgery, Shanghai Sixth People’s Hospital, Shanghai Jiao Tong University School of Medicine, Shanghai, 200233 China; 2 State Key Laboratory of Pharmaceutical Biotechnology, School of Life Sciences, Nanjing University, Nanjing, 210008 Jiangsu China

**Keywords:** Gastric cancer, Traditional Chinese medicine, Graph representation learning, Virtual screening

## Abstract

Traditional Chinese Medicine (TCM) represents a multi-component therapeutic system with substantial chemical complexity. This complexity makes it difficult to directly elucidate anti-gastric cancer mechanisms from macroscopic herbal formulae. To support the systematic prioritization of potential small-molecule candidates, this study focuses on screening bioactive small-molecule constituents from TCM. We constructed a heterogeneous network integrating Chinese herbal pieces (CHPs), Chinese patent medicines (CPMs), genes, diseases, and small molecules, and incorporated metapath2vec representations and attention mechanisms into a graph neural network framework, the model is designed to learn relational patterns among heterogeneous nodes. Network pharmacology and in vitro validation in AGS and MKN-45 gastric cancer cell lines show that Icaritin and Arundine inhibit cell proliferation and induce apoptosis. This study presents an AI-assisted pipeline for candidate prioritization, providing methodological support for the systematic prioritization and preliminary validation of bioactive TCM molecules in gastric cancer.

## Introduction

Gastric cancer (GC) is the fifth most prevalent cancer globally and the fourth leading cause of cancer-related death [1, 2]. Despite advances in chemotherapy, targeted therapy, and immunotherapy, the treatment of gastric cancer continues to face significant challenges, including substantial adverse effects, pronounced tumor heterogeneity, and the development of therapeutic resistance. Therefore, there is a need to develop safer and more effective therapeutic strategies for gastric cancer.

Traditional Chinese Medicine (TCM) provides a rich source of natural products, some of which have been reported to exhibit favorable pharmacological activity and acceptable safety profiles. Natural products derived from TCM represent a valuable yet underexplored source for anticancer drug discovery. Accumulating evidence indicates that TCM-derived compounds and natural products can inhibit tumor cell proliferation, migration, and invasion, induce apoptosis and autophagy, and enhance the response of drug-resistant cancer cells to chemotherapy [3]. In recent years, several monomer compounds derived from TCM have entered clinical trials or received regulatory approval, suggesting promising anticancer potential [4, 5]. For instance, Evodiamine, a natural quinolone alkaloid monomer compound, extracted from Evodia rutaecarpa (Wu-Chu-Yu), can induce apoptosis in gastric cancer cells by inhibiting the FAK/AKT/mTOR signaling pathway and suppress cell proliferation through G2/M cell cycle arrest [6–8]. Piperine, an alkaloid extracted from Piper nigrum (Hu-Jiao), inhibits gastric cancer cell proliferation by modulating the PI3K/AKT pathway [9]. Beyond direct cytotoxic effects, these natural products can also serve as adjuvant therapies. For example, quercetin, a compound commonly found in plants, enhances the inhibition of metastatic capacity in human AGS cells when combined with the frontline gastric cancer drug Irinotecan and its active metabolite SN-38 [10].

However, the TCM system is inherently complex, encompassing multi-component, multi-target, and multi-pathway interactions. This complexity makes it challenging to systematically characterize the molecular targets and mechanisms of action of both herbal formulas and individual herbs, posing significant obstacles for mechanism-guided candidate discovery.

Conventional research in traditional medicine typically adopts a top-down strategy, deconstructing formulae into herbs and their corresponding ingredients. However, this approach is time-consuming and often requires substantial additional experimental validation to elucidate the underlying mechanisms [11]. Recent advances in artificial intelligence have enabled data-driven drug discovery [12, 13]. On the one hand, network-based methods integrated with artificial intelligence can model the synergistic effects of herbal formulae, which helps to elucidate multi-component and multi-target mechanisms [14–17]. On the other hand, knowledge graph-based approaches can integrate large-scale associations among compounds, targets, diseases, and herbal resources, supporting the prioritization of potential bioactive small molecules before experimental validation.

Current computational strategies for drug–disease association prediction primarily include matrix factorization, random walk-based representation learning, and deep learning [18]. Random walk-based methods are widely used for node embedding [19]. Methods such as DeepWalk [20] and node2vec [21] are designed for homogeneous graphs, while metapath2vec extends this paradigm to heterogeneous graphs by incorporating structural constraints such as meta-paths. This enables metapath2vec to capture semantic correlations between different node types while preserving structural information, providing an interpretable foundation for node representation in complex biomedical networks[22]. Among deep learning approaches, graph neural networks (GNNs) demonstrate significant advantages in modeling complex network structures, particularly when processing biomedical data with multiple node types and multi-relational architectures [18]. These advantages have led to the increasing application of GNN models in biomedical knowledge graphs. For example, TxGNN is trained on medical knowledge graphs and integrates GNN with metric learning modules to rank potential indications and contraindications across 17,080 diseases, which enables zero-shot prediction [23]. MRDDA predicts potential drug–disease associations by integrating GNN with graph attention mechanisms [24]. In the field of TCM, network pharmacology is widely used to construct component–target–disease networks to systematically elucidate the synergistic mechanisms of multi-component and multi-target actions. In parallel, several studies apply GNNs to predict associations involving small molecules or herb–disease pairs. HGHDA utilizes a dual-channel hypergraph convolutional network to predict herb–disease associations, while HDCTI predicts drug–target interactions through hypergraph representations [25, 26].

These advances are supported by the growing availability of TCM knowledge resources. Databases such as HERB2.0 [27, 28], SymMap2.0 [29], and ETCM2.0 [30] integrate multidimensional information including herbs, ingredients, targets, and diseases, providing a comprehensive foundation of node and relational data for the construction of knowledge graphs in TCM. However, despite the availability of such rich data resources, existing knowledge graph-based approaches still face several challenges. In particular, drug–disease association data are inherently incomplete, and the absence of confirmed negative samples leads to a positive–unlabeled setting, which may introduce uncertainty into model training and evaluation. In addition, many existing methods are evaluated under edge-level splitting schemes, which may overestimate model performance and limit the ability to generalize to unseen nodes in practical applications.

In this study, we focus on TCM-oriented small-molecule discovery by constructing a large-scale heterogeneous knowledge graph that integrates Chinese patent medicines (CPM), Chinese herbal pieces (CHP), compounds, genes, and diseases, thereby providing a comprehensive representation of TCM-related entities and their interactions. Building on this graph, we develop a graph-based model that combines metapath2vec and GNN with attention mechanisms to capture high-order correlation patterns and support the prioritization of TCM-derived small molecules. We further assess its generalization ability under a node-level splitting setting, which better reflects prediction scenarios involving previously unseen nodes. The predicted candidates are subsequently evaluated through in vitro cellular experiments to assess their biological activity. This framework provides a scalable and interpretable strategy for deconvoluting complex TCM systems and facilitates the precision discovery of bioactive compounds for cancer therapy.

## Materials and methods

### Heterogeneous network construction and feature representation

We integrate multi-source data from TCM-MKG [31] and ChEMBL [32, 33] to construct a heterogeneous information network (HIN), denoted as $\mathcal {G} = (\mathcal {V}, \mathcal {E})$. Here, $\mathcal {V}$ denotes the node set and $\mathcal {E}$ denotes the edge set. The node set contains five node types: disease (*D*), compound (*C*), gene (*G*), Chinese patent medicine (*CPM*), and Chinese herbal pieces (*CHP*). The edge set includes both inter-type and intra-type relationships. Inter-type relationships include $D\text {--}C$, $D\text {--}G$, $D\text {--}CPM$, $C\text {--}G$, $C\text {--}CHP$, and $CPM\text {--}CHP$, while intra-type relationships are constructed within the corresponding node categories. Data preprocessing is conducted using R version 4.3.2, and the graph is implemented using the Deep Graph Library (DGL).

To capture biological and chemical regularities, intra-type similarities are binarized into discrete edges. For disease nodes, semantic similarity $S_{DO}$ is computed using the DOSE package, and an edge is established if $S_{DO} \ge 0.8$. For *CPM* and *CHP*, Jaccard indices based on shared herbal components and chemical constituents are used to construct similarity edges with thresholds of 0.85 and 0.1, respectively. Compound–compound edges are derived from precomputed chemical similarity relationships, and gene–gene edges are obtained from curated protein–protein interaction data.

### Feature encoding

Node features are constructed according to node types. For compound nodes, 1024-dimensional molecular fingerprints are used to represent chemical structure. For disease nodes, precomputed semantic embeddings of dimension 1024 are used. A unified feature space of dimension 2048 is defined as $\textbf{f} \in \mathbb {R}^{2048}$.

The feature vectors for compound and disease nodes are defined as

$$ \textbf{f}_{i}^{C} = [\textbf{m}_i \parallel \textbf{0}_{1024}], \quad \textbf{f}_{j}^{D} = [\textbf{0}_{1024} \parallel \textbf{s}_j], $$

where $\textbf{m}_i \in \mathbb {R}^{1024}$ denotes the molecular fingerprint of compound node *i*, and $\textbf{s}_j \in \mathbb {R}^{1024}$ denotes the disease embedding of node *j*.

For gene, Chinese patent medicine, and Chinese herbal piece nodes, attribute features are first constructed as multi-hot vectors based on domain-specific annotations. These features are then transformed into 2048-dimensional representations during data preprocessing to align with the unified feature space. The resulting 2048-dimensional vectors are used as fixed node attributes in the model.

### Model architecture

The final model is a heterogeneous GCN framework that integrates metapath2vec-based structural initialization, node-type-specific attribute projection, heterogeneous message passing, subnetwork refinement, and attention-based representation fusion.

We first obtain structural embeddings using metapath2vec. Structural embeddings are learned for compound and disease nodes based on metapath-guided random walks under a skip-gram objective. The resulting embeddings for compound and disease nodes are denoted as $\textbf{e}_i^{C} \in \mathbb {R}^{64}$ and $\textbf{e}_j^{D} \in \mathbb {R}^{64}$, respectively. The metapath used in this study is defined as

$$ D \rightarrow C \rightarrow D, $$

which captures disease–compound co-occurrence patterns in the heterogeneous network.

Raw attribute features are projected into the hidden space in a node-type-specific manner. For node type *t*, the projected attribute representation of node *i* is defined as

$$ \textbf{x}_i^{t} = \textbf{W}_{t}^{x}\textbf{f}_i^{t} + \textbf{b}_{t}^{x}, $$

where $\textbf{f}_i^{t}$ is the raw attribute feature of node *i*, and $\textbf{W}_{t}^{x}$ and $\textbf{b}_{t}^{x}$ are learnable projection parameters.

Heterogeneous message passing is performed using relation-specific GraphConv layers. Let $\textbf{h}_i^{(l)}$ denote the representation of node *i* at layer *l*. The update is defined as

$$ \textbf{h}_i^{(l)} = \sigma \left( \sum _{r \in \mathcal {R}} \textbf{W}_r^{(l)} \cdot \textrm{MEAN}_{j \in \mathcal {N}_r(i)} \textbf{h}_j^{(l-1)} + \textbf{W}_0^{(l)} \textbf{h}_i^{(l-1)} \right) , $$

where $\mathcal {R}$ is the set of relation types, $\mathcal {N}_r(i)$ denotes the neighbors of node *i* under relation *r*, $\textbf{W}_r^{(l)}$ and $\textbf{W}_0^{(l)}$ are learnable weight matrices, and $\sigma$ is the activation function. Two GraphConv layers are used.

After heterogeneous propagation, a subnetwork encoder is applied as an additional refinement branch. Multiple relation-specific subnetworks are constructed from selected edge-type subsets, including subnetworks centered on compound–disease, CPM–disease, CPM–CHP, CHP–compound, compound–gene, and gene–disease relation groups. Each subnetwork is encoded separately to produce an additional representation for the node types involved in that subnetwork. The resulting subnetwork representation for node *i* of type *t* is denoted as $\textbf{u}_i^{t}$.

The final node representation is obtained by attention-based fusion over multiple representation sources, including the projected attribute representation, the metapath2vec embedding, the heterogeneous GraphConv representation, and the subnetwork representation. Let $\textbf{z}_{i,m}^{C}$ and $\textbf{z}_{j,m}^{D}$ denote the *m*-th representation source for compound node *i* and disease node *j*, respectively. Separate attention modules are used for compound and disease nodes:

$$ \textbf{h}_i^{*,C} = \sum _{m=1}^{M} \beta _m^{C} \textbf{z}_{i,m}^{C}, \quad \textbf{h}_j^{*,D} = \sum _{m=1}^{M} \beta _m^{D} \textbf{z}_{j,m}^{D}. $$

The final node representations are subsequently used for disease–compound association scoring. The hidden feature dimension is set to 64, and the dropout rate is set to 0.6 in the final implementation.

### Dataset partition

To evaluate generalization under a disease-level node split, we partition diseases rather than randomly splitting compound–disease edges. Positive supervision signals are collected from ChEMBL and consist of 39,675 known compound–disease associations [32, 33]. These diseases are partitioned into training, validation, and test sets at a ratio of approximately 7:1:2. All compound–disease pairs associated with a given disease inherit the same split label, ensuring that diseases in the validation and test sets are excluded from supervised training.

Since experimentally verified non-associations are not available, negative pairs are constructed from unlabeled compound–disease pairs after excluding all known positives. We adopt a two-strategy negative sampling framework, in which negative pairs are generated using either random sampling or structure-based sampling. In the random protocol, negative pairs are sampled uniformly from unlabeled compound–disease pairs within the corresponding disease split. In the structure-based protocol, negative sampling is conditioned on the target disease. For each disease, candidate negative compounds are restricted to unlabeled pairs that remain structurally connected to that disease in the heterogeneous network. Specifically, a compound is included in the candidate set if it shares at least one gene neighbor with the disease through the compound–gene and disease–gene relations, or if it is connected to at least one CHP that links, through CPM, to the disease via the compound–CHP, CHP–CPM, and disease–CPM relations. Negative pairs are then sampled from this disease-specific candidate set after excluding all known positive associations. If no such candidates are available, compounds from matched compound-degree buckets are used as a fallback, and uniform random sampling is applied only when both structure-based and degree-matched candidates are unavailable.

For model development, training and validation are performed under the structure-based protocol with a positive-to-negative ratio of 1:10. For evaluation, both the random and structure-based protocols are applied to the held-out diseases, and each protocol is evaluated under positive-to-negative ratios of 1:1, 1:5, and 1:10. These settings provide complementary views of model performance under different negative sampling distributions and allow assessment of how evaluation metrics vary under matched class ratios with different negative construction rules.

### Model training and link prediction

To prevent information leakage, all compound–disease associations involving validation or test diseases are excluded from the training graph prior to model fitting. The model is trained on the remaining graph using the structure-based 1:10 negative sampling protocol, and validation is performed on the held-out validation diseases under the same setting.

Given a compound–disease pair (*i*, *j*), the model outputs a logit score $s_{ij}$, which is transformed into a probability using the sigmoid function:

1 $$\begin{aligned} \hat{y}_{ij} = \frac{1}{1 + \exp (-s_{ij})}. \end{aligned}$$

Here, $s_{ij}$ denotes the predicted logit for compound *i* and disease *j*, and $\hat{y}_{ij}$ denotes the corresponding association probability.

The model is optimized using Binary Cross-Entropy with Logits Loss. To address the class imbalance introduced by negative sampling, the positive class is weighted according to the negative-to-positive ratio in the training data. Early stopping is based on validation loss, and the test sets are reserved exclusively for final evaluation.

Model performance is evaluated using AUROC, AUPRC, accuracy, precision, recall, and F1-score. AUPRC is treated as the primary metric due to its sensitivity to class imbalance, while AUROC is reported as a complementary measure of ranking performance.

### Candidate filtering and ranking strategy

After model inference, all candidate compound–disease pairs are assigned prediction scores and ranked according to their predicted associations with gastric cancer. To focus on compounds with potential relevance to TCM, we restrict the candidate set to compounds linked to CHP in the heterogeneous network.

To further enrich for natural products, the herb-related candidates are cross-referenced with HERB 2.0 and NPASS, and only compounds supported by both resources are retained. Compounds with previously reported therapeutic relevance to gastric cancer, identified through database annotations and literature searches, are then excluded to prioritize less characterized candidates for downstream validation. Based on the final ranking results, five compounds are selected for subsequent experimental validation.

### PPI network construction and pathway enrichment analysis

The predicted compound targets derived from the compound—gene edges were intersected with gastric cancer–related genes obtained from the GeneCards database (relevance score $\ge$ 30). The overlapping genes were used for subsequent analyses.

A protein–protein interaction (PPI) network was constructed using the STRING database (version 11.5) based on the overlapping targets. The resulting network was imported into R for visualization.

For functional annotation, Gene Ontology (GO) and KEGG pathway enrichment analyses were performed using the clusterProfiler package in R. Gene symbols were converted to Entrez IDs prior to enrichment analysis. The enrichGO function was used for GO enrichment (including BP, CC, and MF categories), and the enrichKEGG function was applied for KEGG pathway analysis.

### Cell lines and culture conditions

The human gastric cancer cell lines AGS and MKN-45, as well as the human embryonic kidney cell line HEK293T, were obtained from Shanghai Zhong Qiao Xin Zhou Biotechnology Co., Ltd. All cell lines were confirmed negative for mycoplasma contamination and authenticated via short tandem repeat (STR) profiling before use.

AGS cells were cultured in Ham’s F-12K (Kaighn’s) medium supplemented with 10% fetal bovine serum (FBS) and 1% penicillin–streptomycin. MKN-45 cells were maintained in RPMI-1640 medium supplemented with 20% FBS and 1% penicillin–streptomycin. HEK293T cells were cultured in DMEM supplemented with 10% FBS and 1% penicillin–streptomycin. All cultures were incubated at 37$\,^\circ \textrm{C}$ in a humidified atmosphere containing 5% CO$_2$.

### Cell viability assay (CCK-8)

AGS, MKN-45, and HEK293T cells are seeded into 96-well plates at a density of $1 \times 10^{4}$ cells per well and allowed to adhere overnight at 37$\,^\circ \textrm{C}$ in a humidified atmosphere containing 5% CO$_2$. The cells are then treated with different concentrations of the tested compounds for 24 h. Subsequently, 10 $\mu$L of CCK-8 solution is added to each well. After incubation for 1.5 h, the optical density at 450 nm is measured using a microplate reader to assess cell viability.

### Apoptosis assay

To assess cellular apoptosis, cells were treated with various concentrations of the specified drugs for 12 h. Following treatment, the cells were detached using EDTA-free trypsin and collected by centrifugation. After being washed twice with ice-cold phosphate-buffered saline (PBS), the cells were resuspended in 300 $\mu$L of binding buffer. Subsequently, 5 $\mu$L of Annexin V-FITC was added and the mixture was incubated for 15 min at room temperature in the dark. Immediately prior to flow cytometric analysis, 5 $\mu$L of Propidium Iodide (PI) was added for nuclear staining. The apoptotic distribution was analyzed using a flow cytometer, and the raw data were processed and visualized using FlowJo software (version 10.8.1) to determine the percentage of early and late apoptotic cells.

### Statistical analysis

All data are presented as mean ± standard deviation (SD) from $$n=3$$ independent biological replicates. Statistical analyses are performed using GraphPad Prism (version 9.5.1). Comparisons between treated groups and the untreated control are conducted using ordinary one-way analysis of variance (ANOVA), followed by Dunnett’s multiple comparisons test. Statistical significance is denoted as $$*P < 0.05$$, $$**P < 0.01$$, and *ns* (not significant). Dose–response curves are fitted using nonlinear regression based on a four-parameter logistic model, and the corresponding $$IC_{50}$$ values are reported with $$95\%$$ confidence intervals. For GO and KEGG enrichment analyses, p-values were adjusted for multiple testing using the Benjamini–Hochberg (BH) method as implemented in the clusterProfiler package. Adjusted p-values (false discovery rate, FDR) were used to assess statistical significance.

## Results

**Fig. 1:**
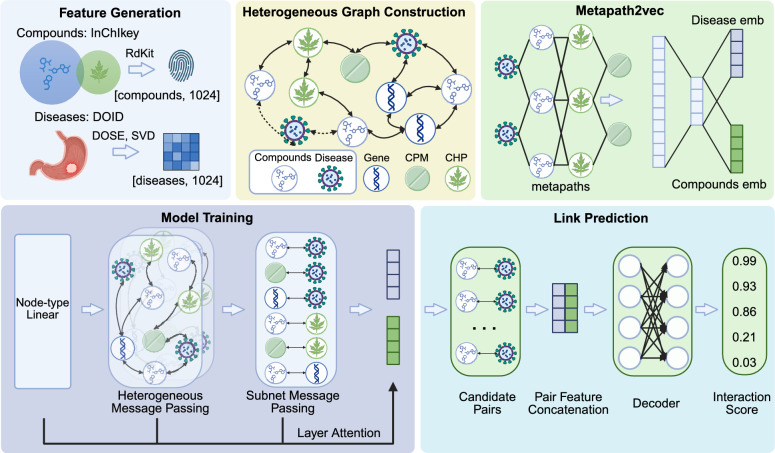
Framework of the neural network-based screening strategy for identifying TCM-derived compounds against gastric cancer

### Framework for virtual screening of candidate drugs for gastric cancer

We establish a systematic computational framework to identify candidate TCM-derived small molecules for gastric cancer (Fig. 1). The framework consists of five stages: feature generation, heterogeneous graph construction, representation learning based on metapath2vec, model training, and link prediction.

In the feature generation stage, compound and disease features are derived from chemical structures and disease ontologies, respectively, while other node types are encoded as multi-hot vectors based on domain-specific attributes. In the graph construction stage, a heterogeneous interaction network (HIN) is built by integrating node and edge information from the ChEMBL [33] and TCM-MKG [31] databases, covering compounds, diseases, genes, CPM, and CHP. In the representation learning stage, metapath2vec is first applied to obtain initial node embeddings through meta-path-guided random walks. In the model training stage, node features are first transformed through node-type-specific linear projections, followed by relation-aware message passing based on heterogeneous GCN layers. A subnetwork message passing module is further introduced to preserve localized interaction patterns within selected relation groups. A layer attention mechanism is then applied to integrate representations from different layers. In the link prediction stage, candidate compound–disease pairs are enumerated, and their representations are concatenated and fed into the decoder to compute interaction scores. These scores are organized into a ranking matrix to prioritize candidate compounds for downstream experimental validation.

### Characterization of the TCM-associated heterogeneous network and molecular chemical space

**Table 1:** Statistics of node and edge types in the heterogeneous network

Category	Type	Number
Node	Compound	106,466
	Disease	9566
	CHP	2606
	CPM	8896
	Gene	19,365
Edge	Compound–Disease	37,203
	Compound–CHP	140,538
	Compound–Gene	2,173,413
	Disease–CPM	16,488
	Disease–Gene	6,411,049
	CHP–CPM	74,084
	Compound–Compound	1,063,116
	Disease–Disease	26,568
	CPM–CPM	18,680
	CHP–CHP	15,204
	Gene–Gene	6,879,008

To ensure the reliability and structural validity of the constructed network, we further performed a quantitative characterization of its topology and the chemical diversity of its components. The fundamental statistics of nodes and edges are summarized in Table 1.

**Fig. 2:**
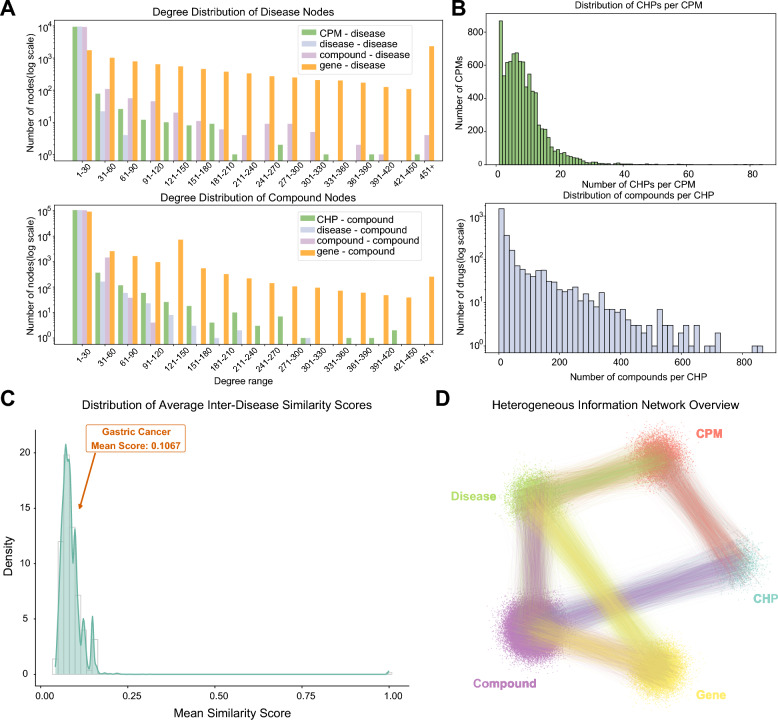
Statistical distribution of the Heterogeneous Information Network. **A** Degree distributions of disease and compound nodes across different relation types. **B** Distributions of the number of CHPs per CPM and compounds per CHP. **C** Distribution of average inter-disease similarity scores across diseases, with the position of gastric cancer indicated. **D** Overview of the heterogeneous information network

Topological analysis shows that the degree distributions of both compound and disease nodes exhibit a long-tailed pattern. This pattern suggest a highly heterogeneous network, in which a small number of hub entities maintain disproportionately large numbers of functional associations (Fig. 2A). For example, while most formulations follow relatively simple compositions, the most complex CPM incorporates over 800 CHP, and certain herbs contain thousands of distinct small molecules. This hierarchical organization reflects the structural complexity of interactions within the TCM system (Fig. 2B).

Building on this structural complexity, we evaluate the position of the target disease within the network. Based on semantic similarity analysis, gastric cancer exhibits a mean similarity score of 0.1067, placing it in a relatively dense region of the similarity distribution (Fig. 2C). This result indicates that gastric cancer shares a certain degree of similarity with other diseases in the network while still maintaining distinct characteristics. Together, these structural and similarity-based properties collectively characterize the global organization of the HIN (Fig. 2D).

**Fig. 3:**
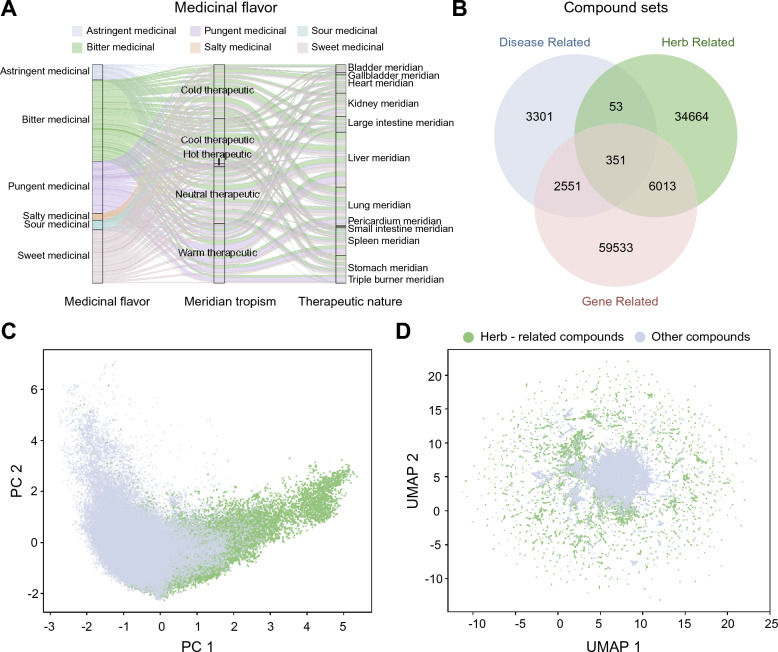
Feature representation and embedding of TCM small molecules. **A** Sankey diagram showing the relationships among medicinal flavors, meridian tropisms, and therapeutic natures. **B** Venn diagram showing the overlap among disease-related, herb-related, and gene-related compound sets. **C** PCA projection of compound features, showing the distribution of herb-related compounds and other compounds in the same feature space. **D** UMAP projection of compound features, illustrating the local distribution patterns of herb-related compounds and other compounds

To connect these network-level characteristics with pharmacological properties, we map the herbal nodes across diverse dimensions, including medicinal flavors, meridian tropism, and therapeutic natures, thereby enriching the network with high-level TCM theoretical descriptors (Fig. 3A).

To further investigate the chemical characteristics of these biological entities, we partition the chemical library into three functional subsets: disease-related, herb-related, and gene-related compounds (Fig. 3B). We observe that a substantial proportion of herb-derived small molecules do not overlap with disease- or gene-associated sets, indicating that many of these compounds are not currently associated with documented clinical indications or annotated molecular targets. To characterize these chemical entities, all compounds are projected into a low-dimensional chemical space using PCA and UMAP. The results show that herb-related compounds and other small-molecule compounds exhibit partial overlap while also displaying distinct distribution patterns in the embedding space. These observations suggest that herb-derived compounds possess chemical features that are not entirely redundant with conventional compound libraries. Accordingly, they may serve as a complementary chemical resource for candidate discovery (Fig. 3C, D).

### Comprehensive evaluation of model performance and robustness across multiple settings

Compound–disease link prediction is conducted under partially observed supervision, where confirmed negative samples are not explicitly available. To enable training and evaluation, negative samples must be constructed. However, their true distribution is not directly observable, and different sampling strategies may lead to varying degrees of class imbalance and potential false negatives.

To account for this uncertainty, we evaluate the model under multiple test settings with different negative sampling ratios and strategies, including balanced (1:1) and imbalanced (1:10) configurations, as well as random and structure-based sampling strategies (Methods). To further assess generalization to unseen disease nodes, we adopt a disease-level node-split protocol, where the supervised compound–disease associations are split according to disease nodes. This setting evaluates whether the model can prioritize candidate compounds for diseases that are not observed during training.

**Fig. 4:**
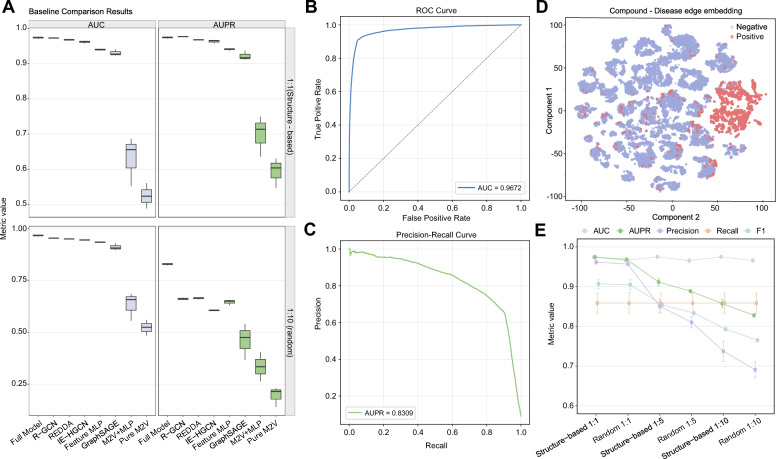
Comprehensive evaluation of model performance under different testing settings. **A** Comparison of the proposed model with baseline methods in terms of AUC and AUPR under both structure-based and random test protocols. **B** Receiver operating characteristic (ROC) curve of the proposed model. **C** Precision–recall (PR) curve of the proposed model. **D** Visualization of compound–disease edge embeddings, where positive and negative samples are projected into a two-dimensional space. **E** Performance of the proposed model under different negative sampling ratios and sampling strategies, including structure-based and random protocols, evaluated using AUC, AUPR, precision, recall, and F1-score

Then we compare the proposed model with both external and internal baselines, including R-GCN [34], REDDA [35], IE-HGCN [36], feature-based MLP, GraphSAGE, metapath2vec+MLP, and pure metapath2vec. The proposed model consistently achieves the best overall performance across the representative test settings (Fig. 4A). Under the 1:10 configuration, the model achieves an AUC of 0.9627 and an AUPR of 0.8309 (Fig. 4B, C). The t-SNE visualization further shows that positive and negative samples occupy distinguishable regions in the embedding space, indicating that the learned representations exhibit separable patterns between positive and negative samples (Fig. 4D).

To further examine the robustness of the model under different sampling conditions, we analyze the effect of varying the negative sampling ratio on evaluation metrics. Increasing the negative sampling ratio mainly reduces AUPR, precision, and F1-score, whereas AUC and recall remain relatively stable across test configurations (Fig. 4E). This decline indicates that a larger negative space increases the difficulty of maintaining a high proportion of true positive associations among top-ranked predictions, which may affect the reliability of candidate prioritization in practical screening scenarios. However, the stable recall suggests that the model maintains its ability to retrieve positive compound–disease associations under varying negative sampling settings.

### Ablation study and interpretability of the predictive framework

**Fig. 5:**
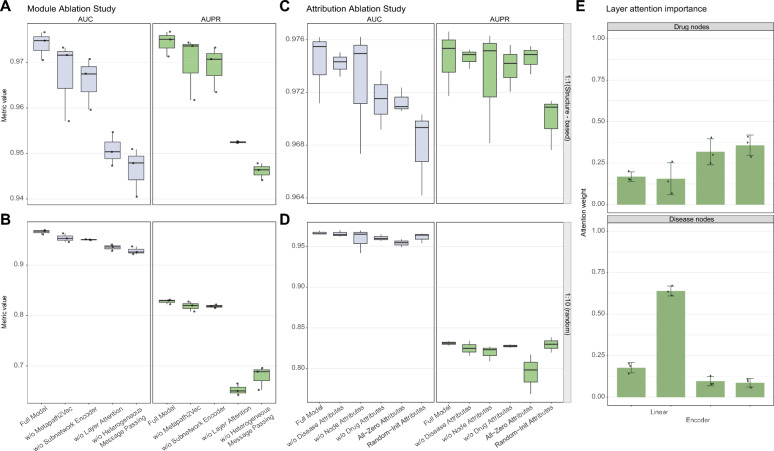
Ablation analysis and interpretability of the proposed model. **A** Module ablation results evaluated by AUC and AUPR under the structure-based test setting. **B** Module ablation results evaluated by AUC and AUPR under the random test setting. **C** Attribution ablation results evaluated by AUC and AUPR under the structure-based test setting. **D** Attribution ablation results evaluated by AUC and AUPR under the random test setting. **E** Layer-wise attention weights for different representation sources in compound and disease nodes

To clarify how each architectural component contributes to prediction performance, we conduct module-level ablation under the same two test settings used above. Metapath2vec, heterogeneous message passing based on heterogeneous GCN, the subnetwork module, and layer-wise attention are removed individually from the full model while keeping other components unchanged. Removing any of these components reduces model performance in both test settings, with more pronounced decreases observed after removing layer-wise attention and heterogeneous message passing (Fig. 5A, B). These results indicate that graph message passing, topological information extraction, and layer-wise selection of informative representations all contribute to compound–disease association prediction.

To determine whether the introduced node attributes provide information beyond graph topology, we further conduct attribute-level ablation. Zero-initialized and randomly initialized attributes yield the weakest performance in both test settings, suggesting that models relying mainly on topology propagation may lack sufficient semantic information (Fig. 5C, D). Removing compound attributes, disease attributes, or attributes from auxiliary node types involved in message propagation also weakens model performance to varying degrees. These findings indicate that the initial semantic features provide complementary information and improve the predictive capacity of the model.

To further interpret these performance differences, we analyze the learned layer-wise attention weights for compound and disease nodes. This analysis reveals which representation layers contribute more strongly to the final prediction. For compound nodes, the subnetwork encoder and heterogeneous message passing layers receive higher attention weights, suggesting that compound representations depend more strongly on graph-derived structural information. In contrast, disease nodes assign the highest attention weight to the node-type linear layer, indicating that disease-specific initial information contributes substantially to their final representations. These attention patterns show that the model adaptively integrates different sources of information for different node types, rather than relying on a single representation layer (Fig. 5E).

### In silico prioritization and target elucidation of TCM-derived anti-gastric cancer candidates

**Fig. 6:**
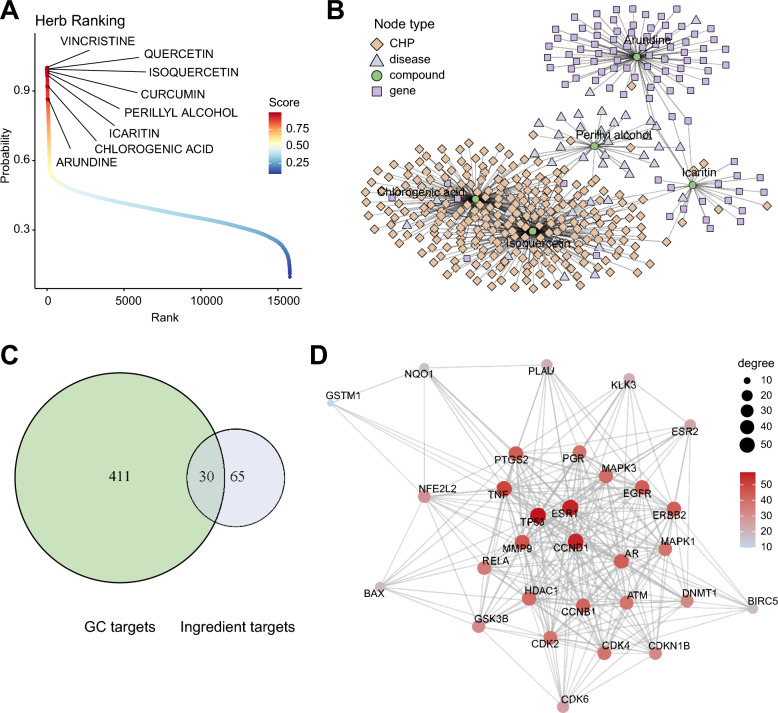
Virtual screening of candidate anti-gastric cancer compounds. **A** Ranking of candidate compounds based on predicted association scores. **B** Network topology of the prioritized compounds and their associated nodes in the heterogeneous network. **C** Venn diagram showing the overlap between gastric cancer-associated genes and predicted targets of the selected compounds. **D** Protein–protein interaction (PPI) network constructed from the overlapping targets

The final model is retrained on the full graph to utilize all available supervised compound–disease associations for candidate prioritization. We then score all candidate compounds for gastric cancer and derive an ingredient-level ranking according to their predicted association scores together with their connections to herbal sources in the network. The ranking results show that several highly ranked compounds, including vincristine, quercetin, isoquercetin, curcumin, perillyl alcohol, icaritin, chlorogenic acid, and arundine, have relatively high prediction scores (Fig. 6A). Some of these compounds have been reported in cancer-related studies but are not explicitly annotated as gastric cancer-associated compounds in the current database, suggesting that the model can recover biologically plausible candidates from incomplete knowledge graphs. Based on the ranking results and literature-based filtering, five compounds are selected for subsequent analysis and experimental validation.

We next examine the heterogeneous topological context of the selected compounds in the network. The local network analysis shows that chlorogenic acid and isoquercetin are connected with multiple herbal sources, whereas icaritin, arundine, and perillyl alcohol show connections with disease- or gene-related neighborhoods (Fig. 6B). These patterns indicate that the selected candidates are supported by different types of evidence in the heterogeneous graph, including herbal occurrence, disease associations, and target-related connections.

To explore target-level associations, we intersect the predicted targets of the selected compounds with gastric cancer-associated genes collected from GeneCards. This analysis identifies 30 overlapping targets between 441 gastric cancer-associated genes and 95 ingredient-associated targets (Fig. 6C). A protein–protein interaction (PPI) network is then constructed for these overlapping targets. Topological analysis highlights TP53, CCND1, ESR1, and MMP9 as high-degree nodes within this network (Fig. 6D).

**Fig. 7:**
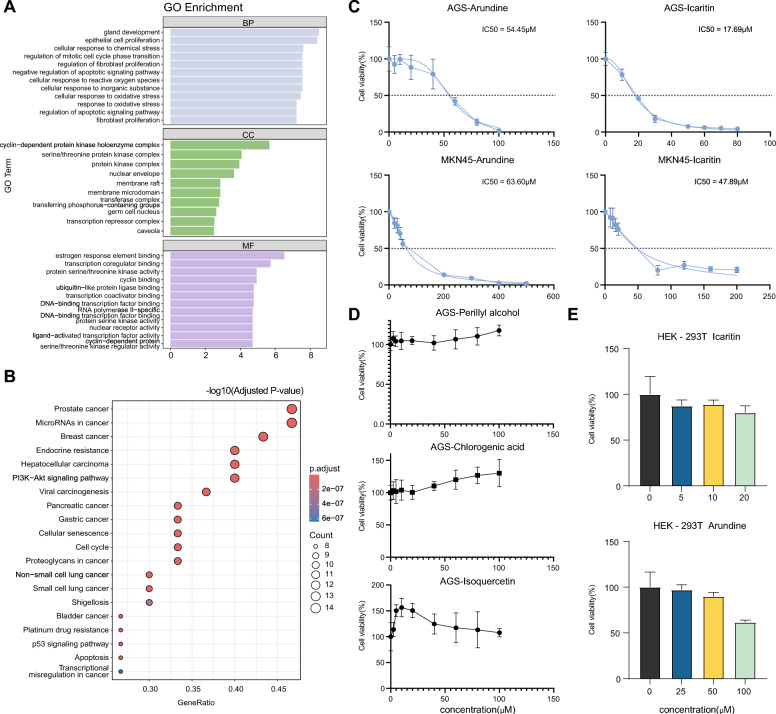
Functional analysis and experimental validation of selected candidate compounds. **A** Gene Ontology (GO) enrichment analysis of overlapping target genes, including biological process (BP), cellular component (CC), and molecular function (MF) categories. **B** KEGG pathway enrichment analysis of overlapping target genes. **C** Dose–response curves and $IC_{50}$ estimation for Arundine and Icaritin in AGS and MKN-45 gastric cancer cell lines. **D** Cell viability of additional candidate compounds in AGS cells across different concentrations. **E** Cell viability of HEK293T cells treated with Icaritin and Arundine. Data are presented as mean ± standard deviation from $n=3$ independent biological replicates

GO enrichment analysis links the overlapping targets to biological processes related to epithelial cell proliferation, gland development, oxidative stress response, and regulation of apoptotic signaling pathways. Enrichment terms related to cell-cycle regulation are also observed, including negative regulation of mitotic cell cycle phase transition (Fig. 7A). KEGG pathway enrichment analysis further shows enrichment in several cancer-related pathways, including gastric cancer, PI3K–Akt signaling, cell cycle, apoptosis, and p53 signaling pathways (Fig. 7B). Together, these enrichment patterns are consistent with cellular processes that are commonly implicated in gastric cancer progression and provide a functional context for the selected candidates.

Together, the ranking, network topology, target overlap, and enrichment analyses indicate that the selected TCM-derived compounds are connected to cancer-related molecular contexts. These computational findings provide a rationale for prioritizing compounds for subsequent in vitro validation.

### Icaritin and Arundine inhibit proliferation and promote apoptotic cell death in gastric cancer cells

We conduct CCK-8 assays to evaluate the effects of the top-ranked candidate compounds on the viability of two gastric cancer cell lines. Arundine and Icaritin reduce cell viability in both AGS and MKN-45 cells across the tested concentrations (Fig. 7C, D). The half-maximal inhibitory concentration ($IC_{50}$) of Arundine is 54.45 $\mu$M in AGS cells and 63.60 $\mu$M in MKN-45 cells. Icaritin shows lower $IC_{50}$ values, with 17.69 $\mu$M in AGS cells and 47.89 $\mu$M in MKN-45 cells. In contrast, the other tested candidates, including perillyl alcohol, isoquercitrin, and chlorogenic acid, do not exhibit a consistent concentration-dependent reduction in cell viability under the tested conditions. These results indicate that the prioritization strategy can identify compounds with measurable anti-proliferative effects in gastric cancer cell lines, although further experimental validation and mechanistic studies are still required. Compounds that do not show clear inhibitory effects in the current CCK-8 assay are not included in subsequent validation experiments under the present experimental conditions.

To further assess the effects of Arundine and Icaritin on non-cancer cells, we additionally evaluate their effects on HEK293T cells using the same CCK-8 assay (Fig. 7E). Under the tested concentration range, both compounds show relatively smaller changes in cell viability compared to gastric cancer cell lines. However, since HEK293T cells are not normal gastric epithelial cells, these results should be regarded as a preliminary reference only and cannot fully reflect the effects of the compounds on normal gastric epithelial cells.

**Fig. 8:**
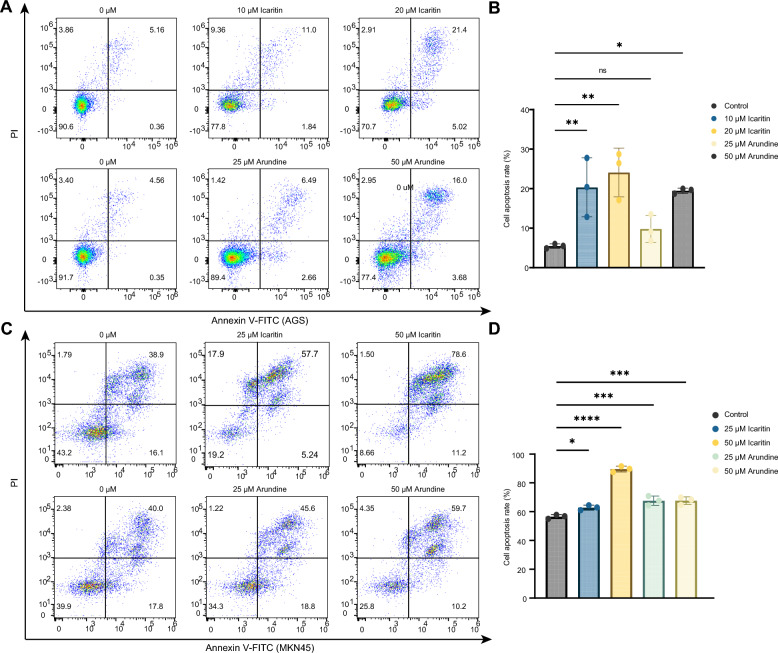
Apoptosis analysis of gastric cancer cells treated with Icaritin and Arundine. **A** Representative flow cytometry plots of AGS cells after 12 h treatment with Icaritin (top row) and Arundine (bottom row). **B** Quantification of apoptosis rates in AGS cells under different treatment conditions. **C** Representative flow cytometry plots of MKN-45 cells after 12 h treatment with Icaritin (top row) and Arundine (bottom row). **D** Quantification of apoptosis rates in MKN-45 cells under different treatment conditions

We further use Annexin V-FITC and PI double staining followed by flow cytometry to evaluate whether Arundine and Icaritin affect cell death in AGS and MKN-45 cells. In AGS cells, treatment with Arundine at 25 and 50 $\mu$M or Icaritin at 10 and 20 $\mu$M increases the proportion of Annexin V-positive apoptotic cells. In MKN-45 cells, both Arundine and Icaritin are tested at 25 and 50 $\mu$M and similarly increase the apoptotic cell population (Fig. 8A–D). These results are consistent with the anti-proliferative effects observed in the CCK-8 assays, suggesting that apoptosis may contribute to the reduced cell viability induced by Arundine and Icaritin. Together, the in vitro results support further investigation of Arundine and Icaritin in gastric cancer models.

## Discussion

This study presents a heterogeneous graph-based computational framework for prioritizing TCM-derived small molecules in gastric cancer. By integrating metapath2vec, heterogeneous message passing, subnetwork encoding, and layer-wise attention, the model integrates multi-relational information across compounds, diseases, genes, CHP, and CPM. The framework shows stable performance under different evaluation settings and provides a practical strategy for narrowing the search space of candidate molecules.

The virtual screening framework prioritizes several compounds with potential relevance to gastric cancer, among which Icaritin and Arundine show measurable biological activity in vitro. Icaritin is a bioactive prenylflavonoid derived from the genus Epimedium. While Icaritin is recognized as an active compound in hepatocellular carcinoma-related studies [37–39], its pharmacological role in gastric cancer is less extensively explored. Arundine, an indole alkaloid found in Arundo donax, has also been reported in cancer-related contexts, including breast cancer [40].

Experimental validation in two gastric cancer cell lines shows that both compounds decrease cell viability in a concentration-dependent manner. Flow cytometry further indicates that treatment with Icaritin or Arundine increases the proportion of apoptotic cells. These findings provide evidence for the biological activity of the prioritized candidates and provide experimental evidence for their further investigation in gastric cancer models.

Several limitations should be considered. First, although the model incorporates disease ontology information, the current disease representation still lacks gastric cancer-specific molecular and clinical descriptors, such as transcriptomic signatures, mutation profiles, pathway activity, and patient-level phenotypes. This limitation may lead the model to prioritize compounds with broad anticancer relevance rather than candidates specific to the pathological context of gastric cancer. Incorporating disease-specific omics data may improve the biological specificity and interpretability of future predictions.

Second, compound–disease association prediction is performed under incomplete supervision, where reliable negative associations are not explicitly available. Although multiple negative sampling settings are used to evaluate robustness, constructed negatives may still contain unrecognized true associations. This uncertainty is inherent to biomedical knowledge graphs and may influence both model training and performance estimation. Future work should further explore uncertainty-aware learning strategies, as well as the curation and construction of higher-confidence negative sample sets, to reduce the effect of incomplete annotations.

Third, the current framework learns predictive associations from graph topology, node attributes, and known interaction patterns, but it does not establish causal drug effects. Although the network analysis suggests several candidate target and pathway axes related to the prioritized compounds, these results should be interpreted as hypothesis-generating evidence rather than experimentally validated mechanisms. The in vitro experiments provide phenotypic validation for selected candidates, but they do not resolve direct targets, upstream regulators, or downstream signaling cascades. Direct validation of the predicted targets and pathways, including protein-level, transcript-level, and perturbation-based assays, remains an important direction for future work. In addition, the current model does not incorporate contraindications, adverse reactions, pharmacokinetic properties, or patient-specific information, which are essential for evaluating translational potential. Future improvements should integrate multi-omics data, safety-related knowledge, and patient-level molecular profiles to support more precise and biologically grounded TCM-derived drug discovery.

Fourth, not all prioritized compounds exhibit measurable biological activity under the current experimental conditions, which highlights the presence of prediction failures. These cases are primarily attributed to modeling-related factors. The framework learns statistical associations from heterogeneous networks, and high-degree hub nodes may introduce bias by disproportionately influencing the learned representations. As a result, compounds connected to hub nodes may be preferentially ranked, even when their functional relevance is limited. Conversely, biologically relevant candidates associated with low-degree nodes may be underrepresented due to insufficient structural evidence. In addition, the model captures association patterns rather than causal therapeutic effects, and high predicted scores may reflect general co-occurrence or shared pathways instead of direct efficacy in gastric cancer. A more systematic analysis of these failure cases, including degree-aware evaluation and causal filtering strategies, may help distinguish between correlation-driven predictions and functionally relevant candidates. Incorporating such analyses may further improve the interpretability and reliability of the prediction framework.

Overall, this study establishes a graph-based computational framework that links TCM knowledge integration, computational candidate prioritization, and experimental screening, providing a practical strategy for discovering bioactive small molecules from complex TCM-derived resources.

## Data Availability

The datasets and source code used in this study are publicly available at https://github.com/naevisaespa/TCM-DrugScreening-GNN.

## Abbreviations

AUC: Area under the curve
AUPRC: Area under the precision–recall curve
AUROC: Area under the receiver operating characteristic curve
BH: Benjamini–Hochberg
ChEMBL: ChEMBL database
CHP: Chinese herbal piece
CPM: Chinese patent medicine
DGL: Deep Graph Library
DOID: Disease Ontology identifier
DOSE: Disease Ontology Semantic and Enrichment analysis
EDTA: Ethylenediaminetetraacetic acid
ETCM: Encyclopaedia of Traditional Chinese Medicine
FDR: False discovery rate
FITC: Fluorescein isothiocyanate
GC: Gastric cancer
GCN: Graph convolutional network
GNN: Graph neural network
GO: Gene Ontology
HERB: High-throughput experiment- and reference-guided database of Traditional Chinese Medicine
HIN: Heterogeneous information network
KEGG: Kyoto Encyclopedia of Genes and Genomes
M2V: Metapath2vec
MLP: Multilayer perceptron
NPASS: Natural Product Activity and Species Source database
PCA: Principal component analysis
PPI: Protein–protein interaction
PR: Precision–recall
R-GCN: Relational graph convolutional network
ROC: Receiver operating characteristic
STR: Short tandem repeat
STRING: Search Tool for the Retrieval of Interacting Genes/Proteins
SVD: Singular value decomposition
t-SNE: t-distributed stochastic neighbor embedding
TCM: Traditional Chinese Medicine
UMAP: Uniform Manifold Approximation and Projection
